# INR-to-platelet ratio (INPR) as a novel noninvasive index for predicting liver fibrosis in chronic hepatitis B

**DOI:** 10.7150/ijms.51799

**Published:** 2021-01-09

**Authors:** Rongrong Ding, Jianming Zheng, Dan Huang, Yanbing Wang, Xiufen Li, Xinlan Zhou, Li Yan, Wei Lu, Zongguo Yang, Zhanqing Zhang

**Affiliations:** 1 Department of Hepatobiliary Medicine, Shanghai Public Health Clinical Center, Fudan University, Shanghai 201508, China.; 2 Department of Integrative Medicine, Shanghai Public Health Clinical Center, Fudan University, Shanghai 201508, China.; 3 Department of Infectious Diseases, Huashan Hospital, Fudan University, Shanghai 200040, China.

**Keywords:** international normalized ratio, INR, platelet, liver fibrosis, chronic hepatitis B

## Abstract

**Objective:** We aimed to investigate whether a novel noninvasive index, i.e., the international normalized ratio-to-platelet ratio (INPR), was a variable in determining liver fibrosis stage in patients with chronic hepatitis B (CHB).

**Methods:** A total of 543 treatment-naïve CHB patients were retrospectively enrolled. Liver histology was assessed according to the Metavir scoring scheme. All common demographic and clinical parameters were analyzed.

**Results:** Based on routine clinical parameters (age, sex, HBeAg status, HBV DNA, hematological parameters, coagulation index, and liver biochemical indicators), a novel index, i.e., the INR-to-platelet ratio (INPR), was developed to magnify the unfavorable effects of liver fibrosis on INR and platelets. The AUCs of INPR for predicting significant fibrosis, advanced fibrosis, and cirrhosis were 0.74, 0.76 and 0.86, respectively. Compared with APRI, FIB-4, and GPR, the INPR had comparable predictive efficacy for significant fibrosis and better predictive performance for advanced fibrosis and cirrhosis.

**Conclusion:** INPR could be an accurate, easily calculated and inexpensive index to assess liver fibrosis in patients with CHB. Further studies are needed to verify this indicator and compare it with other noninvasive methods for predicting liver fibrosis in CHB patients.

## Introduction

Chronic hepatitis B (CHB) is a potentially life-threatening healthcare issue and a major cause of liver cirrhosis and hepatocellular carcinoma 1, 2. It is estimated that there are more than 240 million people with CHB worldwide, and approximately 780,000 people die from complications of hepatitis B every year, including cirrhosis, hepatic failure and hepatocellular carcinoma 1. Based on early diagnosis and effective antiviral therapy, the prognosis of CHB can be significantly improved, even if it histologically manifests as advanced fibrosis or cirrhosis 3. Thus, early staging of hepatic fibrosis and assessing risk in CHB patients are of great significance.

Liver biopsy is still regarded as the gold standard for the assessment of inflammation and fibrosis. However, it has some limitations, such as invasiveness, sampling variability, and associated risk of complications 4. Additionally, it is difficult to dynamically follow up on the progression of liver fibrosis. Therefore, several noninvasive methods for evaluating liver fibrosis have been developed to minimize the need for liver biopsies and their drawbacks. Among these markers, the aspartate aminotransferase-to-platelet ratio index (APRI) 5, 6 and FIB-4 score 7 are the two most widely used methods. Although they have been recommended for the diagnosis of cirrhosis in resource-limited settings by the WHO guidelines 1, their value for assessing CHB patients remains in dispute 5, 8, 9. GPR has been proposed as a new routine method to identify patients with severe fibrosis or cirrhosis in CHB patients 10; however, diagnostic values have varied among studies 11, 12.

Blood platelet count is a simple and inexpensive parameter in routine clinical practice. Previous studies have reported that platelet counts decrease with liver fibrosis progression and have demonstrated their potential predictive performance in liver fibrosis for CHB patients 13, 14. Kim HJ *et al.* 15 reported that the international normalized ratio (INR) was increased in liver cirrhosis. Another study found that INR was correlated independently and significantly with liver fibrosis in chronic hepatitis C patients 16. Recently, a study by Farid K *et al.* 17 demonstrated that prothrombin-INR scores contributed to predicting the occurrence of large esophageal varices in patients with hepatitis C virus-induced liver cirrhosis. Therefore, the INR could be considered a potential indicator of liver fibrosis.

Whether combining INR and platelet count can increase the predictive value for liver fibrosis is an attractive issue. In this study, we developed a novel and simple index, the INR-to-platelet ratio (INPR), in CHB patients and compared the diagnostic value of the INPR with that of the APRI, FIB-4, and GPR.

## Materials and Methods

### Ethics Statement

The study protocol and informed consent documents were reviewed and approved by the Ethics Committee of Shanghai Public Health Clinical Center, Fudan University. All CHB patients provided written informed consent during their admission.

### Patients

Between January 2016 and October 2019, a total of 543 consecutive anti-HBV naïve patients with CHB who had undergone liver biopsies at Shanghai Public Health Clinical Center, Fudan University, were retrospectively enrolled in the study. The inclusion criteria were persistently having serum hepatitis B surface antigens for more than 6 months 18 and a liver biopsy within one week of the blood laboratory examinations. The exclusion criteria were hepatocellular carcinoma, antiviral treatment history, decompensated cirrhosis, inadequate liver biopsy samples (<1.5 cm), coinfection with other types of viral hepatitis, history of overt alcohol consumption (>40 g/d), autoimmune liver disease, hereditary metabolic liver disease, and use of anticoagulant drugs.

### Data collection

Percutaneous liver biopsy was performed using 16-G needle under ultrasound guidance. Liver samples with a minimum length of 1.5 cm and at least 7 complete portal tracts were fixed in 10% formalin, embedded in paraffin, and stained with HE, Masson's trichrome and reticulin for histological analysis.

Fasting blood samples were obtained within one week of liver biopsy. Platelets and other blood cells were counted using a Sysmex-XT 4000i automated hematology analyzer. INR and other coagulation indices were measured using a STAR Max automatic coagulation analyzer. ALT, AST and other serum biochemical parameters were measured using an Architectc16000 automatic biochemical analysis system.

The formulas for APRI, FIB-4, and GPR are as follows: APRI = (AST (U/L)/ULN of AST)/platelet count (10^9^/L) × 100; FIB-4 = (age (years) × AST(U/L))/(platelet count (10^9^/L) × (ALT (U/L)) ^1/2^); and GPR = (GGT (U/L)/ULN of GGT)/platelet count (10^9^/L) × 100.

### Outcomes

Liver histology was analyzed by two experienced pathologists who were blinded to other laboratory data according to the Metavir scoring system 19, as follows: F1 (portal fibrosis without septa), F2 (portal fibrosis with rare septa), F3 (numerous fibroses without cirrhosis), and F4 (cirrhosis). In this study, liver fibrosis stages F2-4 were defined as significant fibrosis, F3-4 were defined as advanced fibrosis, and F4 was defined as cirrhosis.

### Statistical analysis

Statistical analysis was performed using IBM SPSS Statistics software, version 26.0 (SPSS Inc., Chicago, IL, USA). Continuous variables are reported as median (interquartile range (IQR)) and were compared by the Kruskal-Wallis test. Categorical variables are reported as proportions and were compared by the chi-square test. Laboratory parameters were classified as binary variables by upper or lower limits of normal. Logistic regression models were used to screen the useful laboratory parameters for assessing liver fibrosis levels. The performances of noninvasive markers for predicting liver fibrosis were evaluated by receiver operating characteristic (ROC) curve analyses using Stata software, version 16.0 (STATA Corp., Texas, USA). A two-sided P<0.05 was considered statistically significant.

## Results

### Baseline characteristics of CHB patients

A total of 543 treatment-naïve patients with CHB were enrolled in the study. The characteristics of all of the recruited patients are summarized in **Table 1**. The patients were a median age of 37 years old and were mostly male (67.4%). Fifty (9.2%) patients had metabolic-associated fatty liver disease (MAFLD); 338 (62.2%) patients were HBeAg positive. The fibrosis stages were 142 (26.2%) in F1, 147 (27.1%) in F2, 91 (16.8%) in F3, and 163 (30.0%) in F4. As the liver fibrosis stage increased, the median levels of ALP, GGT, DBil, globulin, total bile acid, and INR increased, while the median levels of cholinesterase, albumin, prealbumin, and platelet counts decreased (all *P* < 0.001).

### A novel index consisting of INR and platelets for predicting liver fibrosis

Variables including age, sex, MAFLD, HBsAg, HBeAg, HBV DNA, ALT, AST, ALP, GGT, TBil, DBil, cholinesterase, albumin, globulin, prealbumin, total bile acid, FBG, TC, TG, HDL, LDL, urea, creatinine, eGFR, INR, WBC, RBC, neutrophils, platelets, and hemoglobin were included in the univariate analysis. The presence of significant liver fibrosis (F2-4) was associated with MAFLD, HBV DNA, ALT, AST, ALP, GGT, DBil, prealbumin, cholinesterase, globulin, total bile acid, TC, TG, LDL, urea, INR, and WBC, platelet, hemoglobin, neutrophil, and RBC counts. Multivariable analysis identified ALT, GGT, prealbumin, INR, and platelets as independent predictors of significant liver fibrosis (**Table 2**). The presence of liver cirrhosis (F4) was associated with age, sex, MAFLD, AST, ALP, GGT, DBil, prealbumin, cholinesterase, total bile acid, TC, LDL, HDL, globulin, INR, and WBC, platelet, hemoglobin, neutrophil, and RBC counts. Multivariable analysis identified albumin, cholinesterase, INR, and platelets as independent predictors of cirrhosis (**Table 3**). Thus, only INR and platelets were independent predictors of significant fibrosis and cirrhosis (all P < 0.05).

**Figure 1:**
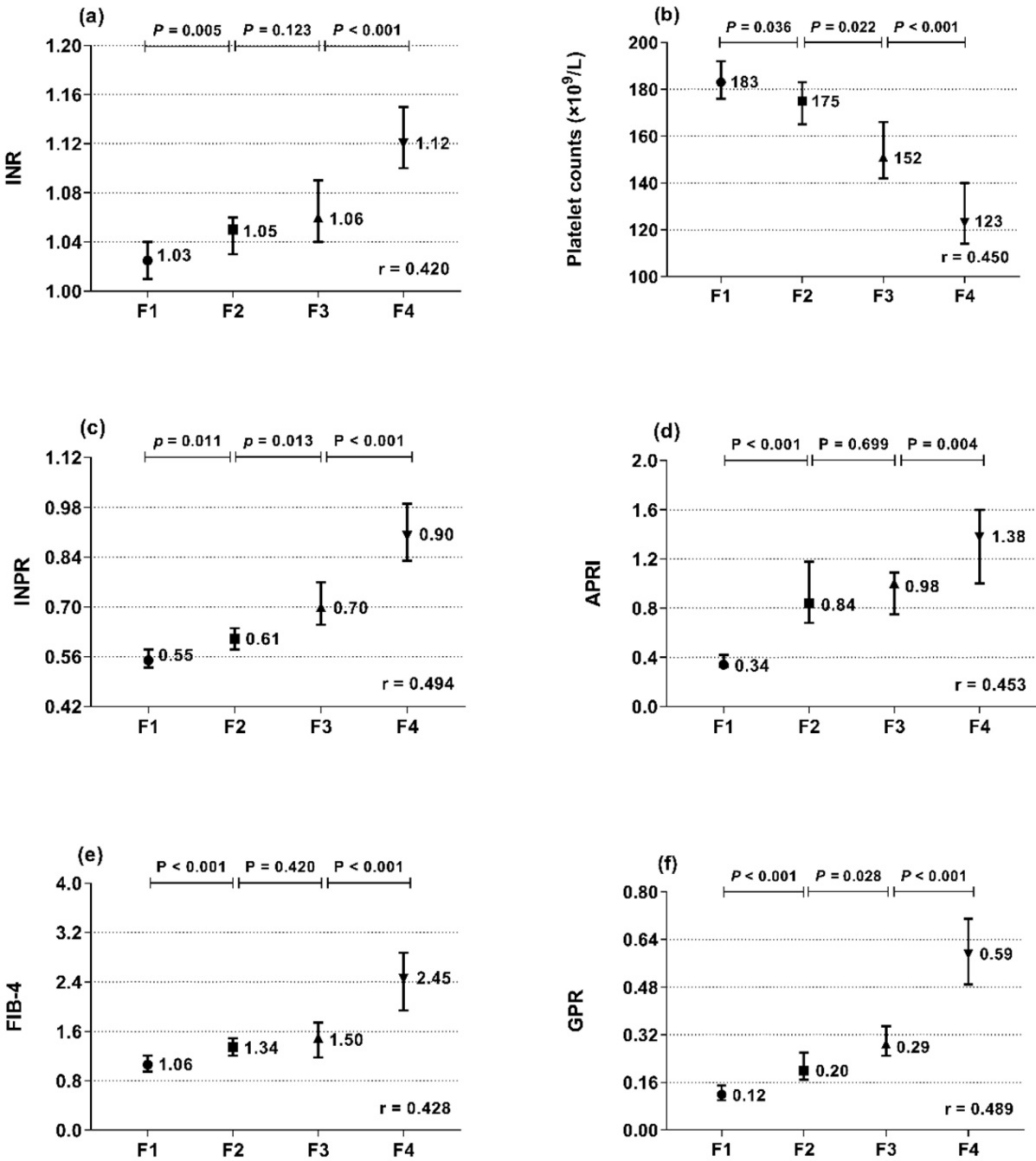
Median and 95% CI of INR (a), platelet count (b), INPR (c), APRI (d), FIB-4 (e), and GPR (f) in the four subgroups (F1, F2, F3, and F4) classified by fibrosis stage (Metavir scores). *r* is the correlation coefficient of the variables with the fibrosis stages.

The associations of INR and platelets with liver histopathology were further analyzed. INR and platelets in relation to the Metavir fibrosis stage are shown in **Figure 1a, b**. INR was positively correlated with the Metavir score (r = 0.420, *P* < 0.001), while platelets were negatively correlated (r = -0.450, *P* < 0.001). Thus, we designed the INR to platelet ratio (INPR) as INR/platelet counts (×10^9^/L)×100 to amplify the difference between the INR and platelets in CHB patients with different stages of fibrosis. INPR was significantly positively correlated with Metavir fibrosis stage with a higher correlation coefficient than GPR, APRI, and FIB-4 (r = 0.494, 0.489, 0.453, and 0.428, respectively) (**Figure 1c-f**).

### Comparison of INPR, APRI, FIB-4, and GPR in predicting significant liver fibrosis, advanced fibrosis, and cirrhosis in patients with CHB

**Figure 2:**
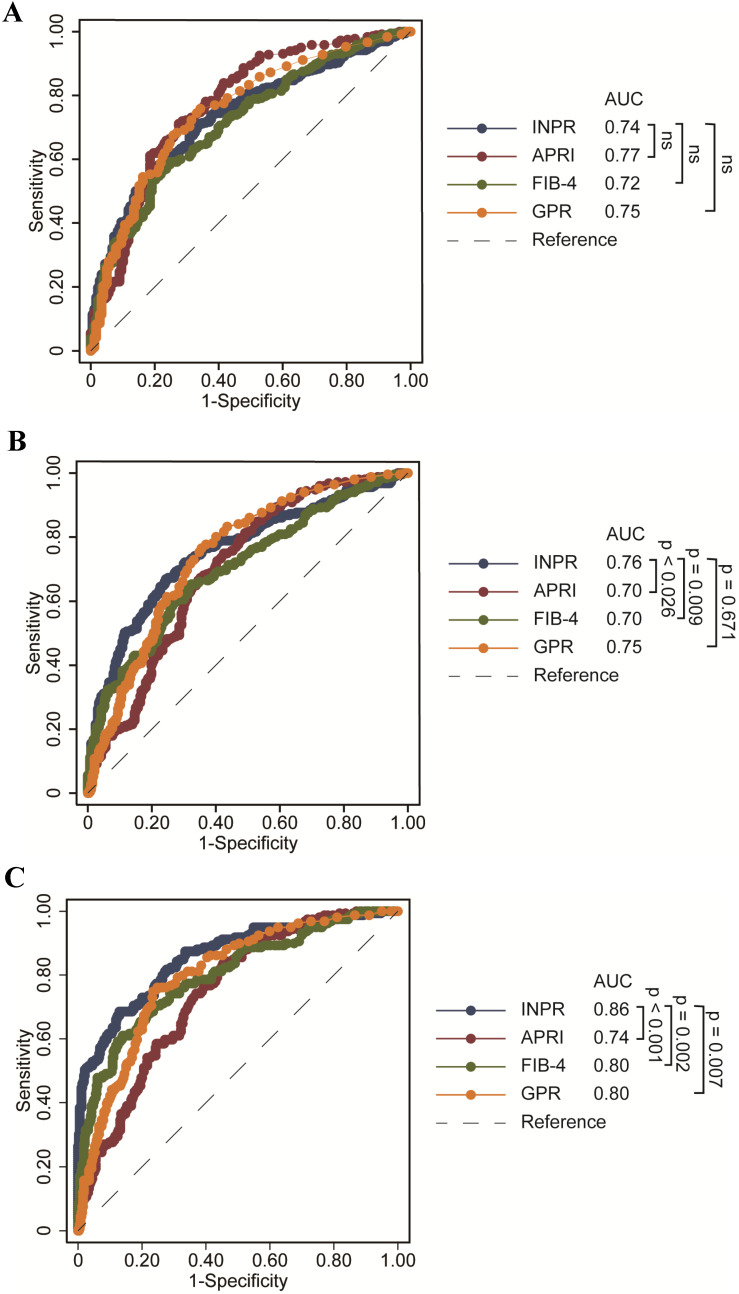
ROC comparison of INPR, APRI, FIB-4, and GPR for predicting significant fibrosis and cirrhosis. (a) ROC comparison for predicting significant fibrosis; (b) ROC comparison for predicting advanced fibrosis; (c) ROC comparison for predicting cirrhosis.

The study calculated the data comparing the fourth serum fibrosis scores related to significant liver fibrosis, advanced fibrosis, and cirrhosis in patients with CHB by ROC curve analysis. The ROC curves of INPR, APRI, FIB-4, and GPR are shown in **Table 4**. In determining significant liver fibrosis (F2-4), the AUROCs of INPR, APRI, FIB-4, and GPR were 0.74 (sensitivity 57.62%, specificity 80.34%), 0.77 (sensitivity 71.15%, specificity 72.63%), 0.72 (sensitivity 56.59%, specificity 78.21%), and 0.75 (sensitivity 74.45%, specificity 68.72%), respectively. When distinguishing advanced liver fibrosis (F3-4), the AUROCs of INPR, APRI, FIB-4, and GPR were 0.76 (sensitivity 67.06%, specificity 75.61%), 0.70 (sensitivity 73.23%, specificity 759.52%), 0.71 (sensitivity 59.84%, specificity 73.36%), and 0.74 (sensitivity 74.80%, specificity 566.44%), respectively. For discriminating cirrhosis (F4), the AUROCs of INPR, APRI, FIB-4, and GPR were 0.86 (sensitivity 68.75%, specificity 87.07%), 0.74 (sensitivity 83.23%, specificity 55.76%), 0.80 (sensitivity 60.87%, specificity 86.13%), and 0.80 (sensitivity 75.16%, specificity 76.70%), respectively. Comparing ROC curves using the DeLong method, the INPR showed comparable performance to GPR, APRI, and FIB-4 for assessing significant fibrosis but significantly better performance for predicting cirrhosis than the other three biomarker panels (all *P* < 0.05, **Figure 2**).

## Discussion

Timely assessment of liver fibrosis stage is extremely important in patients with CHB 20. Early diagnosis and continuous follow-up of liver fibrosis progression are essential to prevent liver cirrhosis and end-stage liver disease. Liver biopsy is still considered the gold standard method to diagnose liver fibrosis stage, but some drawbacks significantly limit its clinical application 21. Therefore, many noninvasive methods and model designs with advantages of convenience and good repetition have been developed to stage liver fibrosis in recent years. Transient elastography (TE), shear wave elastography (SWE), and magnetic resonance elastography (MRE) have been reported to have good performances in predicting liver fibrosis stage 22-24. However, they are expensive, and available equipment is lacking in resource-limited areas. Serum markers such as APRI, FIB-4 and GPR have been applied to noninvasively predict liver fibrosis stage 25-27. However, the sensitivity and specificity of diagnosis by these markers have been limited 28.

In this retrospective study, we developed a simple and inexpensive marker consisting of INR and platelet count to predict liver fibrosis in patients with CHB. Prothrombin time-INR increases along with the progression of liver fibrosis, while platelet counts decrease, and this abnormal condition is especially obvious in patients with cirrhosis 29, 30. Liang *et al.* 31 reported that INR and platelet counts were independent predictors of cirrhosis in patients with CHB. In the study by Kayadibi *et al.* 32, the AUROC of platelet count for determining significant liver fibrosis was 0.827 in patients with chronic HCV. Thrombocytopenia in liver disease is due to the accumulation and destruction of platelets in portal hypertensive splenomegaly, arising from progressive liver fibrosis and partly due to impaired production of thrombopoietin in cirrhosis 33, 34. In the present study, the INPR significantly increased as the liver fibrosis stage increased, while hepatic function, such as cholinesterase, albumin, and prealbumin, significantly decreased as liver fibrosis progressed. By ROC analysis, the INPR showed good performance for staging significant fibrosis (F2-4), with an AUROC of 0.74. Notably, the performance was excellent for predicting advanced fibrosis (F3-4) and cirrhosis (F4), with AUROCs of 0.76 and 0.86, respectively. In comparison with APRI, FIB-4, and GPR, the performance of the INPR showed larger AUROCs for diagnoses of both F3-4 and F4, suggesting that it sufficiently reflected the amount of accumulated fibrosis tissue in the liver.

APRI and FIB-4 are the two most widely studied noninvasive serum models, and they have been recommended for detecting liver fibrosis and cirrhosis in resource-limited settings by the WHO HBV guidelines due to their very low cost, easy access, and regular use testing order in clinical practice, and they have been substantially used to identify liver fibrosis and cirrhosis 1. Although previous studies have demonstrated that APRI and FIB-4 had good performance for the identification of significant fibrosis and cirrhosis 35, 36, their accuracy is disputed in patients with CHB 25, 26. Some studies have shown that APRI and FIB-4 evaluated liver fibrosis in CHB patients with moderate accuracy, but they might not be ideal measures to predict liver fibrosis 6, 37. In our study, APRI exhibited AUROCs of 0.77 for predicting significant fibrosis and 0.74 for detecting cirrhosis, similar to previous studies 38-40. The performance of FIB-4 was relatively moderate for the prediction of F2-4, with an AUROC of 0.72. In line with the results from previous studies, FIB-4 showed good performance for detecting cirrhosis, with an AUROC of 0.80 38.

GPR is a new noninvasive marker for liver fibrosis in patients with chronic HBV infection despite the diagnostic value of GPR being atypical 10, 11, 41-43. Our findings demonstrated the good performance of GPR in predicting significant fibrosis, advanced fibrosis and cirrhosis, with AUROCs of 0.75, 0.74, and 0.80. This outcome matched with a previous study that showed that GPR was a promising predictor of significant fibrosis and cirrhosis 10, 42. Recent studies 44, 45 have analyzed the diagnostic accuracy of GPR according to HBeAg status. In the studies by Dong *et al.* 44 and Peng *et al.* 45 for HBeAg-positive CHB, GPR was better than APRI in predicting advanced fibrosis and cirrhosis but was comparable to FIB-4 in distinguishing significant fibrosis, advanced fibrosis and cirrhosis; however, for HBeAg-negative CHB, the predictive performance of GPR in assessing significant fibrosis, advanced fibrosis and cirrhosis was comparable to that of FIB-4 and APRI. Therefore, HBeAg status is not the main factor leading to the difference. We believe that differences in basic characteristics, sample size, spectrum bias of fibrosis distribution, HBV genotypes and different histological scoring systems will lead to differences in results.

By optimized cutoff values of the INPR, significant fibrosis, advanced fibrosis, and cirrhosis could be accurately diagnosed in 64.9%, 71.4% and 81.3% of CHB patients, respectively. The diagnostic accuracies of APRI, FIB-4, and GPR for advanced fibrosis were 65.5%, 66.6%, and 69.6%, respectively. The diagnostic accuracies of APRI, FIB-4, and GPR for cirrhosis were 67.4%, 78.4%, and 76.1%, respectively. The current results indicated that the INPR showed better diagnostic accuracy than the other three noninvasive markers in predicting advanced fibrosis and cirrhosis.

This study had some limitations. First, it was a retrospective study, which might essentially have incurred selective bias. Thus, the diagnostic performance of INPR must be confirmed in prospective studies. Second, we cannot compare the performance of FibroScan to the INPR because of the lack of TE data for most enrolled patients.

In conclusion, our study indicated that the INPR could be a potentially useful, easily calculated and inexpensive index to predict liver fibrosis in patients with CHB, especially in resource-limited settings. Further studies are needed to verify this indicator and compare it with other noninvasive methods for predicting liver fibrosis in CHB patients.

## Figures and Tables

**Table 1:** Baseline characteristics of enrolled patients with chronic hepatitis B

Variables	Total ( n = 543)	F1 (n = 142)	F2 (n = 147)	F3 (n = 91)	F4 (n = 163)	P value
Age, years	37 (31-46)	39 (30-49)	36 (30-43)	36 (31-46)	39 (32-47)	0.145
Male, n (%)	366 (67.4)	96 (67.6)	93 (63.3)	63 (69.2)	114 (69.9)	0.333
MAFLD, n (%)	50 (9.2)	25 (17.6)	10 (6.8)	6 (6.6)	9 (5.5)	0.001
Log_10_[HBsAg], IU/ml	3.55 (3.21-4.10)	3.55 (3.06-4.12)	3.86 (3.41-4.35)	3.52 (3.11-3.95)	3.45 (3.09-3.72)	< 0.001
HBeAg positive, n (%)	338 (62.2)	76 (53.5)	106 (72.1)	62 (68.1)	94 (57.7)	0.004
Log_10_ [HBVDNA], IU/ml	6.33 (4.19-7.310	4.67 (2.01-6.70)	7.02 (4.96-7.67)	6.28 (4.74-7.21)	6.25 (4.54-7.10)	< 0.001
ALT, U/L	64.00 (32.00-157.00)	34.00 (18.75-72.75)	94.00 (43.00-183.00)	79.00 (39.00-211.00)	67.00 (38.00-177.00)	< 0.001
AST, U/L	46.00 (27.00-95.00)	27.00 (19.00-40.50)	53.00 (31.00-113.00)	53.00 (31.00-102.00)	59.00 (35.00-117.00)	< 0.001
ALP, U/L	80.00 (65.00-101.00)	71.00 (59.00-85.00)	77.00 (63.00-97.00)	81.00 (66.00-97.00)	97.00 (76.00-119.00)	< 0.001
GGT, U/L	41.00 (21.00-85.00)	21.00 (15.00-45.25)	35.00 (18.00-89.00)	45.00 (26.00-85.00)	67.00 (37.00-123.00)	< 0.001
TBil, μmol/L	15.60 (11.40-21.80)	14.60 (11.30-18.00)	14.40 (10.60-19.40)	14.70 (11.10-19.80)	18.20 (13.00-28.00)	< 0.001
DBil μmol/L	5.80 (4.30-8.40)	5.20 (4.00-6.43)	5.50 (4.00-7.40)	5.60 (4.30-8.20)	7.50 (5.20-15.10)	< 0.001
Cholinesterase, U/L	7391.00 (6002.00-8787.00)	9072.00(7520.00-10493.50)	7505.00 (6501.50-8550.50)	7361.50 (6344.00-8323.25)	5994.00 (4491.00-7328.00)	< 0.001
Albumin, g/L	41.70 (38.60-44.40)	43.05 (41.00-46.00)	42.00 (38.90-44.40)	41.20 (39.10-44.60)	39.00 (35.10-43.00)	< 0.001
Globulin, g/L	30.00 (26.83-33.00)	29.00 (25.00-31.00)	30.00 (27.00-32.00)	29.00 (27.00-32.00)	31.00 (27.00-35.00)	< 0.001
Prealbumin, g/L	185.00 (131.00-241.88)	249.70 (204.75-291.65)	177.00 (131.00-221.63)	174.00 (138.50-215.08)	148.00 (100.00-203.64)	< 0.001
Total bile acid, μmol/L	9.70 (4.60-18.95)	4.90 (2.80-9.13)	9.10 (4.65-17.55)	10.40 (5.45-15.70)	17.90 (9.58-45.38)	< 0.001
FBG, mmol/L,	4.79 (4.47-5.20)	4.91 (4.65-5.28)	4.77 (4.46-5.20)	4.76 (4.49-5.13)	4.71 (4.34-5.13)	0.007
TC, mmol/L	4.14 (3.65-4.81)	4.32 (3.87-5.05)	4.20 (3.71-4.85)	4.10 (3.61-4.87)	3.87 (3.42-4.56)	< 0.001
TG, mmol/L	0.94 (0.72-1.28)	1.00 (0.81-1.50)	0.91 (0.70-1.24)	0.91 (0.71-1.22)	0.93 (0.71-1.26)	0.023
HDL, mmol/L	1.31 (1.03-1.62)	1.27 (1.02-1.57)	1.39 (1.12-1.67)	1.36 (1.09-1.68)	1.19 (0.95-1.63)	0.001
LDL mmol/L	2.59 (2.10-3.17)	2.75 (2.29-3.48)	2.59 (2.19-3.21)	2.55 (2.07-3.03)	2.39 (1.99-2.90)	< 0.001
Urea, mmol/L	310.25 (253.00-365.40)	328.00 (262.00-382.00)	293.00 (243.10-369.10)	322.00 (262.75-364.25)	303.50 (252.75-341.62)	0.032
Creatinine, μmol/L	65.20 (54.95-74.70)	64.10 (56.60-74.15)	63.40 (54.08-74.90)	67.75 (54.90-73.78)	65.70 (54.50-75.85)	0.819
eGFR, ml/(min×1.73m^2^)	116.42 (102.71-133.15)	115.55 (101.90-133.70)	120.55 (102.82-135.01)	112.42 (102.75-130.67)	116.57 (102.89-133.42)	0.537
INR	1.06 (1.01-1.13)	1.03 (0.98-1.07)	1.05 (1.00-1.10)	1.06 (1.01-1.14)	1.12 (1.06-1.22)	< 0.001
WBC count, ×10^9^/L	5.24 (4.22-6.22)	5.58 (4.64-6.63)	5.09 (4.34-6.01)	5.13 (4.22-6.12)	4.85 (3.91-6.11)	0.003
RBC count, ×10^9^/L	4.61 (4.23-4.98)	4.81 (4.44-5.10)	4.60 (4.29-4.98)	4.55 (4.26-4.95)	4.48 (3.97-4.91)	< 0.001
Platelet count, ×10^9^/L	159.00 (126.00-196.00)	183.00 (156.75-216.00)	175.00 (147.00-201.00)	152.00 (126.00-192.00)	123.00 (89.00-157.00)	< 0.001
Neutrophils count, ×10^9^/L	2.81 (2.16-3.54)	3.21 (2.55-3.96)	2.72 (2.26-3.42)	2.64 (2.11-3.49)	2.53 (1.93-3.30)	< 0.001
Hemoglobin, g/L	145.00 (130.00-155.00)	147.50 (133.75-157.00)	145.00 (131.00-156.00)	146.00 (131.00-155.00)	140.00 (125.00-154.00)	0.036

MAFLD, metabolic associated fatty liver disease; FBG, fasting blood glucose; TC, total cholesterol; TG, total triglycerides; HDL, high density lipoprotein; LDL, low density lipoprotein; eGFR, estimated glomerular filtration rate; WBC, white blood cell; RBC, red blood cell.

**Table 2:** Univariate and multivariate analyses of clinical items and significant fibrosis in patients with CHB (n=364)

Variables	Significant fibrosis (F2-4)			
	Univariate		Multivariate	
	OR (95% CI)	*P* value	OR (95% CI)	*P* value
MAFLD, yes vs. no	0.32 (0.18-0.57)	< 0.001	----	----
Log_10_ [HBVDNA], IU/ml	1.32 (1.19-1.46)	< 0.001	----	----
ALT, U/L	1.00 (1.00-1.01)	< 0.001	0.99 (0.99-0.99)	0.002
AST, U/L	1.01 (1.00-1.01)	< 0.001	----	----
ALP, U/L	1.01 (1.01-1.02)	<0.001	----	----
GGT, U/L	1.01 (1.00, 1.01)	< 0.001	1.02 (1.01-1.04)	< 0.001
DBil, umol/L	1.09 (1.04-1.14)	< 0.001	----	----
Prealbumin, g/L	0.98 (0.98-0.99)	< 0.001	0.99 (0.98-0.99)	< 0.001
Cholinesterase, U/L	1.00 (0.99-1.00)	< 0.001	----	----
Globulin, g/L	1.08 (1.03-1.12)	< 0.001	----	----
Total bile acid, µmol/L	1.04 (1.02-1.06)	< 0.001	----	----
TC, mmol/L	0.65 (0.52-0.80)	< 0.001	----	----
TG, mmol/L	0.53 (0.38-0.76)	< 0.001	----	----
LDL, mmol/L	0.57 (0.45-0.72)	< 0.001	----	----
Urea, mmol/L	0.99 (0.99-1.00)	0.040	----	----
INR	2.57 (1.99-3.32)	< 0.001	**1.75 (1.20-2.59)**	**0.005**
WBC count, ×10^9^/L	0.79 (0.71-0.90)	< 0.001	----	----
Platelet count, ×10^9^/L	0.99 (0.98-0.99)	< 0.001	**0.99 (0.98-0.99)**	**0.005**
Hemoglobin, g/L	0.98 (0.97-0.99)	0.015	----	----
Neutrophil count, ×10^9^/L	0.61 (0.51-0.73)	< 0.001	----	----
RBC count, ×10^12^/L	0.44 (0.31-0.62)	< 0.001	----	----

Variables including age, sex, MAFLD, HBsAg quantity, HBeAg status, HBVDNA, ALT, AST, ALP, GGT, TBil, DBil, prealbumin, albumin, cholinesterase, total bile acid, FBG, TC, TG, LDL, HDL, urea, creatinine, eGFR, INR, WBC, RBC, platelet, neutrophil, and hemoglobin were included in the univariate model. Only variables significantly associated with significant fibrosis in univariate analysis were presented and enrolled in multivariate model.

**Table 3:** Univariate and multivariate analyses of clinical items and cirrhosis in patients with CHB (n=161)

Variables	Cirrhosis (F4)			
	Univariate		Multivariate	
	OR (95% CI)	*P* value	OR (95% CI)	*P* value
Age, years	1.02 (1.00-1.04)	0.014	----	----
Gender, male	1.55 (1.03-2.33)	0.036	----	----
MAFLD, yes vs. no	0.36 (0.16-0.82)	0.014	----	----
AST, U/L	1.00 (1.00-1.00)	0.001	----	----
ALP, U/L	1.01 (1.00-1.02)	< 0.001	----	----
GGT, U/L	1.00 (1.00-1.01)	0.001	----	----
DBil, µmol/L	1.05 (1.03-1.07)	< 0.001	----	----
Prealbumin, g/L	0.99 (0.99-1.00)	< 0.001	----	----
Albumin, g/L	0.84 (0.80-0.88)	< 0.001	0.89 (0.83-0.97)	0.006
Cholinesterase, U/L	0.99 (0.99, 1.00)	< 0.001	1.00 (0.99-1.00)	0.001
Total bile acid, µmol/L	1.02 (1.01, 1.03)	< 0.001	----	----
TC, mmol/L	0.57 (0.45-0.73)	< 0.001	----	----
LDL, mmol/L	0.59 (0.45-0.77)	< 0.001	----	----
HDL, mmol/L	0.45 (0.27-0.73)	0.001	----	----
Globulin, g/L	1.08 (1.04-1.12)	< 0.001	----	----
INR	3.15 (2.46-4.04)	< 0.001	**2.52 (1.72-3.68)**	**< 0.001**
WBC count, ×10^9^/L	0.78 (0.69-0.90)	< 0.001	----	----
Platelet count, ×10^9^/L	0.97 (0.96-0.97)	< 0.001	**0.97 (0.96-0.98)**	**< 0.001**
Hemoglobin, g/L	0.99 (0.98-1.00)	0.045	----	----
Neutrophil count, ×10^9^/L	0.69 (0.57-0.84)	< 0.001	----	----
RBC count, ×10^12^/L	0.47 (0.33-0.65)	< 0.001	----	----

Variables including age, sex, MAFLD, HBsAg quantity, HBeAg status, HBVDNA, ALT, AST, ALP, GGT, TBil, DBil, prealbumin, albumin, cholinesterase, total bile acid, FBG, TC, TG, LDL, HDL, urea, creatinine, eGFR, INR, WBC, RBC, platelet, neutrophil, and hemoglobin were included in the univariate model. Only variables significantly associated with cirrhosis in univariate analysis were presented and enrolled in multivariate model.

**Table 4:** Predictive values of INPR, APRI, FIB-4 and GPR for liver fibrosis in CHB patients

	F2-4 (n = 364)		F3-4 (n = 254)		F4 (n = 161)	
	Estimate	95%CI	Estimate	95%CI	Estimate	95%CI
**INPR**						
AUROC	0.74	0.70-0.78	0.76	0.72-0.79	0.86	0.83-0.90
Cutoff	0.69	-	0.72		0.83	-
Sen (%)	57.62	52.3-62.8	67.06	60.9-72.9	68.75	61.0-75.8
Spe (%)	80.34	73.7-85.9	75.61	70.2-80.5	87.07	83.3-90.3
PPV (%)	85.6	81.3-89.0	70.7	65.9-75.1	69.2	62.9-74.8
NPV (%)	48.3	44.8-51.8	72.3	68.4-75.9	86.8	83.9-89.3
DA (%)	64.9		71.4		81.3	
**APRI**						
AUROC	0.77	0.73-0.80	0.70	0.66-0.74	0.74	0.71-0.78
Cutoff	0.66	-	0.69		0.69	-
Sen (%)	71.15	66.2-75.8	73.23	67.3-78.6	83.23	76.5-88.6
Spe (%)	72.63	65.5-79.0	59.52	53.6-65.2	55.76	50.6-60.8
PPV (%)	84.10	80.5-87.1	61.4	57.6-65.1	44.2	41.0-47.5
NPV (%)	55.30	50.7-59.8	71.7	66.9-76.0	88.7	84.7-91.8
DA (%)	71.6		65.5		67.4	
**FIB-4**						
AUROC	0.72	0.68-0.75	0.71	0.67-0.74	0.80	0.76-0.83
Cutoff	1.56	-	1.65		2.33	-
Sen (%)	56.59	51.3-61.8	59.84	53.5-65.9	60.87	52.9-68.5
Spe (%)	78.21	71.4-84.0	73.36	67.9-78.4	86.13	82.2-89.4
PPV (%)	84.1	79.8-87.6	66.4	61.4-71.0	64.9	58.3-71.0
NPV (%)	47.0	43.5-50.5	67.5	63.8-71.0	83.9	81.1-86.4
DA (%)	63.3		66.6		78.4	
**GPR**						
AUROC	0.75	0.70-0.78	0.74	0.71-0.78	0.80	0.76-0.83
Cutoff	0.20		0.25		0.38	
Sen (%)	74.45	69.6-78.9	74.80	69.0-80.0	75.16	67.7-81.6
Spe (%)	68.72	61.4-75.4	66.44	60.7-71.9	76.70	72.1-80.9
PPV (%)	82.9	79.4-85.8	66.2	62.1-70.0	57.6	52.6-62.5
NPV (%)	56.9	52.0-61.8	75.0	70.5-79.0	88.0	84.8-90.6
DA (%)	72.0		69.6		76.1	

Sen, sensitivity; Spe, specificity; PPV, positive predictive value; NPV, negative predictive value; DA, diagnostic accuracy.
